# Evaluation of the Implementation of an Outreach Clinic for Opioid Use Disorder: Protocol for a Participatory Cocreation and Implementation Study

**DOI:** 10.2196/72457

**Published:** 2025-09-18

**Authors:** Andrée-Anne Paré-Plante, Laurence Fortin, David-Martin Milot, Catherine Langlois, Charlotte Payette-Toupin, Bao Duyen Angéline Nguyen, Sophie Poulin, Karine Bertrand, Caroline Leblanc, Lara Maillet, François Racine-Hemmings, Isabelle Wilson, Christine Loignon

**Affiliations:** 1 Department of Family Medicine and Emergency Medicine Université de Sherbrooke Sherbrooke Canada; 2 Community Health Science Department Université de Sherbrooke Sherbrooke, QC Canada; 3 École Nationale d'Administration Publique Québec Canada

**Keywords:** opioid use disorders, people who use drugs, people who inject drugs, participatory research, outreach clinic, low-threshold treatment program, primary care, photovoice

## Abstract

**Background:**

The opioid overdose crisis currently affecting Canada has resulted in thousands of deaths, and the COVID-19 pandemic has exacerbated the consequences of this crisis, particularly through the instability of the unregulated drug market. The province of Quebec is observing a similar pattern: the opioids consumed are more dangerous, and the number of overdoses is rising. Opioid use disorder (OUD) therefore represents a major public health issue. Offering appropriate interventions, such as opioid agonist therapy integrated into primary care, is one strategy to reduce the risk of death from overdose.

**Objective:**

The aim of this research is to evaluate the implementation of an outreach clinic offering a low-threshold treatment program for OUD in Quebec. The secondary objective is to identify the factors that foster the participation in primary care research of people who are socially excluded and have current or past lived experience of OUD.

**Methods:**

This study is being conducted in the Montérégie region of Quebec and comprises 3 phases: exploratory, photovoice, and participatory evaluation. The qualitative research adopts a participatory approach by involving people who are socially excluded and targeted by the outreach clinic’s services (eg, people experiencing homelessness and living with OUD). A committee of peer researchers, made up of experts with current or past lived experience of OUD, will be set up and will hold 10 meetings at various stages of the research. Two participant profiles will be involved: (1) health care professionals and community workers, who will take part in semistructured interviews; and (2) people with current or past lived experience of OUD, who will take part in the photovoice sessions or peer researcher committee meetings.

**Results:**

The peer researcher committee was formed in winter 2024, and 10 meetings had been held as of June 2025. As of August 2025, 4 photovoice sessions had been conducted, and 14 health care professionals and community workers had participated in the semistructured interviews. This study was funded in September 2022, with funding available through March 2025. Data were collected from September 2022 through June 2025. The analysis was finished in spring 2025. Results of the study are expected to be published in winter 2026.

**Conclusions:**

The anticipated outcome is the establishment of an outreach clinic for OUD outside a major urban center, with a range of services tailored to the needs of people who are socially excluded and living with OUD. The coconstruction of this clinic in collaboration with people with current or past lived experience of OUD will enable an adequate response to the targeted population’s overall health needs and help reduce the barriers to access that they may face in conventional care structures.

**International Registered Report Identifier (IRRID):**

DERR1-10.2196/72457

## Introduction

### Background

Over the last few years, the prevalence of opioid abuse has increased in North America [1], and tens of thousands of people have died from opioid overdoses in the United States [2] and Canada [3]. Since the global COVID-19 pandemic, Canada has experienced a worsening opioid crisis [4,5]. Fentanyl and the emergence of fentanyl analogs—extremely potent opioids with a high risk of overdose—contribute to this situation [3,6]. In Canada, an 89% increase in deaths from these substances was reported between April and December 2020 compared with the same period the previous year [3,7]. Since this marked increase, the number of deaths per quarter has continued to fluctuate around the 2020 peak and could be even higher in 2024 [7]. In the province of Quebec, the situation follows a similar pattern: the opioids consumed are more dangerous, and the number of overdoses is rising [6]. Problematic opioid use also involves other risks that should not be overlooked [8,9], such as the sharing of injection equipment among people who inject drugs, which is associated with an increased risk of blood-borne infectious diseases such as HIV, hepatitis B, and hepatitis C [9-11]. The overdose crisis in North America is mainly driven by the introduction of synthetic opioids into the unregulated drug market [12]. Managing the opioid crisis, and more specifically opioid use disorder (OUD), is a major public health issue in Canada [13], including within Quebec [6,14-16], given that OUD is a chronic, recurrent disorder with personal, societal, and economic consequences that are preventable through appropriate primary care interventions [17-19].

### Populations That Are Socially Excluded and Access to Opioid Agonist Therapy

Management of OUD must include opioid agonist therapy (OAT) using buprenorphine-naloxone or methadone, which should be offered as first- and second-line treatment, respectively [18,20-23]. OAT has been shown to reduce cravings associated with opioid use, lower the risk of opioid overdose, and significantly improve quality of life for people with OUD [17]. However, few people with OUD have access to OAT in Quebec [17]. In Montreal, the most recent statistics on local services indicated that, in 2016, the targeted coverage rate for people with OUD was 70%, while the actual coverage rate was only 36% [24]. This discrepancy is associated with the fact that populations that are socially excluded, such as people who inject drugs and people experiencing homelessness and living with OUD, often encounter obstacles in accessing services due to the high threshold requirements of OAT programs (eg, abstinence from drug use and complex administrative procedures), which do not match the needs and realities of these individuals [24-26].

### Accessibility of Services Outside Major Urban Centers

The opioid overdose crisis is not confined to the downtown areas of major urban centers [27,28]. The same progression is observed all over Canada, particularly in the Montérégie region, the second most populous administrative region in Quebec [29], where deaths increased during the COVID-19 pandemic [30]. One of the particularities of this mixed urban-rural region close to the Montreal metropolis is that, while the needs of people experiencing OUD are very much present, programs offering OAT, as well as adapted addiction treatment programs for people who are socially excluded, are scarce, with most addiction treatment programs concentrated in downtown Montreal (A-A Paré-Plante and Y Chertouk, unpublished data, 2017). This concentration forces people with OUD to move to the metropolis, where exposure to an environment unfavorable to their condition (eg, availability of substances) could exacerbate it [31]. Having to leave their community to receive care increases the precariousness of their situation [27,28]. Moreover, a survey by the Direction de la santé publique (directorate of public health) of the Montérégie region regarding fatal overdoses recommends creating user committees to develop new awareness approaches that would better reflect the on-the-ground reality of people who use drugs [30]. Montérégie faces a lack of services, particularly in the spheres of community resources, mental health, and postdetoxification transition, which considerably compromises continuity of care [32].

### An Outreach Clinic to Improve Access to Services

An outreach clinic offering a low-threshold treatment program for OUD has been established in the Longueuil area, located in the Montérégie region. The opioid crisis and the current situation in Montérégie make it necessary to evaluate the implementation of such a clinic, coconstructed with users and adopting a holistic approach to meeting their needs. It also responds to the need to improve overall health care and services for people in the most vulnerable situations in and around Longueuil and, indeed, elsewhere in Quebec and Canada. This need has been described on several occasions [32]. The results obtained will enable us to improve access to health and social care and services on an ongoing basis according to the needs expressed by the target population. As such, this intervention and project will improve health equity, a benefit that aligns with the United Nations’ Sustainable Development Goal 3 of ensuring well-being and healthy lives for all.

Montérégie’s addiction treatment programs do not adequately meet the needs of its community, and we have little data on participatory evaluations of implementations similar to this intervention. This study will support the evaluation of the implementation of an outreach clinic outside the major Canadian centers (Montreal, Vancouver, or Toronto), coconstructed by and for people who are socially excluded and living with OUD.

### Research Objectives

The aim of this project is to support a participatory evaluation of the implementation of an outreach clinic offering a low-threshold treatment program for OUD that opened in Longueuil in 2022. The secondary objective is to identify the factors that foster the participation in primary care research of people who are socially excluded and have current or past lived experience of OUD.

## Methods

### A Participatory Approach

This research project is based on a participatory approach that promotes the active and voluntary involvement of people who are socially excluded (eg, people experiencing homelessness, people who use drugs, and people who inject drugs) at all stages of the research process [33]. The populations for whom health services are intended should be at the heart of the implementation process [34]. The participatory approach allows for the genuine inclusion of people who are socially excluded. In this study, it will also foster partnership between researchers and community members while stimulating knowledge development and empowerment [35-37]. Moreover, participatory methods contribute to enhancing the scientific quality and transferability of research [37].

This research approach promotes the active involvement of all stakeholders, including people who are socially excluded, in the health care system throughout the research process [38,39]. It fosters the codevelopment of new knowledge and innovative practices that integrate academic and experiential knowledge and reflect the real needs of the community [38-42]. It enables the inclusion in the research process of the traditionally excluded voices of people in the most vulnerable situations [42-44], thereby fostering their empowerment and presenting them with the opportunity to exert an influence on the care and services that concern them [40,45]. Such involvement also helps align health and social services research with the needs expressed by the community [39,40]. Community-based research enables the development of initiatives that improve the quality-of-care delivery and diversify the range of services offered to better meet community needs [42,45,46]. This protocol has been developed in accordance with qualitative study guidelines and the ObsQual checklist for qualitative protocols [47].

### Study Population and Sampling

Three different groups of participants will be involved. Group 1 (n=15-20) will consist of health care professionals and community workers, recruited through convenience sampling in connection with their work at the outreach clinic and partner organizations. Group 2 (n=10) will consist of people who are socially excluded and whose profiles correspond to those of the outreach clinic’s users (eg, people experiencing homelessness and living with OUD who would otherwise be in an addiction treatment program in Longueuil). These participants will also be recruited through convenience sampling, with the help of the Direction de la santé publique of the Montérégie region and partner community organizations. Group 3 (n=4) will consist of members of the peer researcher committee, who have current or past lived experience of OUD and homelessness or other marginalized conditions. They will be recruited directly by the research team.

### Participant Selection Criteria

The inclusion criteria for health care professionals and community workers are as follows: participants must be adults, able to provide consent, and able to speak either English or French. In addition, they must be working in services or within a community organization that serves people who use the outreach clinic.

The inclusion criteria for photovoice participants and members of the peer researcher committee are as follows: participants must be adults, able to provide consent, and able to speak either English or French. They must also meet the *Diagnostic and Statistical Manual of Mental Disorders, Fifth Edition* criteria for OUD (current or past). Furthermore, their profile should correspond to that of the outreach clinic’s target population for OUD.

There are no exclusion criteria.

### Data Collection

This study comprises three phases: (1) exploration with partners and creation of a committee of peer researchers with current or past lived experience of OUD, (2) photovoice with people who are socially excluded and living with OUD, and (3) participatory evaluation of the clinic.

#### Phase 1: Exploration With Partners and Creation of the Peer Researcher Committee

Phase 1 involves 2 preliminary meetings with the collaborators involved in setting up the outreach clinic. These preparatory meetings are not part of the project but will be used to determine research methods, analyses, and the knowledge transfer strategy. The committee of peer researchers with current or past lived experience of OUD (n=4) was formed to reflect the timeline under the leadership of 2 citizen partners (peer researchers: n=4) and will meet approximately once a month for 2 hours to discuss various topics and carry out project-related tasks.. The planned schedule of meeting topics is detailed in Multimedia Appendix 1. The peer researcher committee is limited to 4 members to ensure active, meaningful, and safe participation by all members and to reflect the team’s capacity to accomplish this objective throughout the study.

#### Phase 2: Photovoice With People Who Are Socially Excluded and Living With OUD

The photovoice process aims to explore how the services implemented when the clinic first opened meet the needs of the population served and to adapt these services to the needs expressed by the population. Photovoice is a visual participatory research method that has been used in a number of health research projects. One advantage of this method is that it encourages introspection, the acquisition of knowledge, and the promotion of critical dialogue. The research question, which will be refined with those involved, is as follows: how do the services provided by the clinic meet the needs of the people it serves? For photovoice participants, this question will be phrased in the first person (eg, How do the services I receive at this clinic meet my needs?).

Using voluntary convenience sampling, we will recruit 10 people who could benefit from the clinic’s services to participate in 5 sessions, as detailed in Textbox 1.

**Photovoice sessions.**

**Session 1**

A preparatory meeting for the photo-taking activity (statement of research question, ethical considerations, and reflections on poverty)

**Sessions 2 and 3**

Discussions on the presentation of 5 photos each, responding to the research question (2 h each), which will be recorded and cofacilitated by an experienced researcher (C Loignon) and a family physician (AAP-P)

**Session 4**

A meeting to validate the data analysis resulting from the discussions, as summarized by the researchers and reviewed by the participants (1 h)

**Session 5**

A meeting to present the results (1 h)

The target number of participants is 10, as the research team believes that this will provide data representative of the people benefiting from the services offered by the clinic, which is expected to eventually serve 100 to 200 people. A peer researcher will be present at the preparatory photo-taking meeting to help participants with their questions and photo-taking needs. Participants are expected to use their smartphones to take photos, but the research team will lend a digital camera to participants who are unable to use a personal device. Over a 4-week period between the first and second meetings, participants may take as many photos as they wish; however, they will select 5 photos to present at the discussion sessions. If they wish, they can be accompanied by a member of the research team during the photo-taking process. The research team’s observations during the photovoice sessions, the data collected in the logbook, and participants’ spontaneous discussions with the peer researchers will help address the secondary research objective: identifying the factors favoring the participation in a research project of people who are socially excluded and have current or past lived experience of OUD.

#### Phase 3: Participatory Evaluation of the Outreach Clinic’s Low-Threshold Treatment Program

A participatory evaluation of the clinic’s low-threshold treatment program will be conducted from the start of the project. The evaluation will use multiple qualitative methods, including field observations and individual semistructured interviews (n=15-20) with health care professionals and community workers, supported by a research logbook of observations of clinic activities and informal interviews with field actors. The sample size for the targeted semistructured interviews is 15 to 20 participants or until data redundancy is achieved [48]. In addition, the research team will conduct meetings with the peer researcher committee on various topics; for example, the committee will validate the interview guide with health care professionals and community workers, provide feedback on the analysis of the interviews, and offer input on the results of the photovoice sessions. Members of the peer researcher committee will also conduct observations over the course of the project. During these sessions, they will observe the facilities, the services offered, the atmosphere in the clinic for both users and professionals, and any other relevant aspects. No confidential information will be collected. The peer researcher committee will analyze these observations and then make recommendations for improvements to the clinic. Finally, based on the results obtained across all phases of the project, the committee will choose a method to disseminate the results it deems relevant and organize the dissemination activity. The interview guide for professionals is provided in Multimedia Appendix 2.

### Data Analysis and Interpretation

A thematic analysis will be conducted iteratively, following the approach formulated by Braun and Clarke [49], to enhance rigor and credibility. Trustworthiness [50] (confirmability, dependability, credibility, and transferability) will be ensured through an audit trail (study logbook), validation of our interpretations with peer researchers, and multidisciplinary peer debriefing sessions to address the researchers’ personal reflexivity and methodological limitations [49,51].

The data will be organized through coding, which will consist of labeling the various elements addressed in the interviews or photovoice discussions. These interviews and discussions will be recorded. Data analysis will be iterative: each interview or discussion (individual or group) will be coded as soon as verbatim transcription is completed, and the data will then be integrated into the research team’s coding tables. NVivo software (Lumivero) will be used to facilitate the data analysis. The validity of the analyses and interpretations will be ensured through triangulation at all stages of the research. Triangulation will also apply to the formulation of hypotheses and the development of conclusions. The findings from interviews and discussions will be triangulated with data from observations, the research team’s logbook, and informal interviews with key actors. To this end, members of the research team, including a peer researcher (C Loignon, AAP-P, and LF), will meet regularly to participate in interpretations and facilitate the analysis of multiple data sources. The peer researcher committee will validate the data interpretations at meetings, where summary tables of the data will be presented. All data collection and analysis procedures will be described in the research reports, enabling a rigorous methodological evaluation.

### Ethical Considerations

#### Ethics Approval

The research protocol was approved by the ethics committee of the Centre intégré en santé et services sociaux de la Montérégie-Centre–Hôpital Charles-LeMoyne (project number: MP-04-2022-698).

#### Informed Consent

A consent form for the research project was created, setting out all risks and benefits of participation. Potential participants will have time to review the form before providing consent and may withdraw from the project at any stage.

#### Privacy and Confidentiality

The interviews and the content of the photovoice discussions will be depersonalized using digital codes for each participant. No recordings will be disseminated outside of the research team. Information that could lead to the identification of participants will be removed during the transcription of the interviews and discussions (individual or group).

#### Compensation Details

To acknowledge the expertise of individuals with lived experience of OUD, homelessness, or other marginalized conditions, participants in the peer researcher committee and the photovoice project will be compensated for their time. Members of the peer researcher committee will receive CAD $40 (US $29.08) in the form of a grocery store gift card and CAD $60 (US $43.62) in cash for each 2-hour meeting, along with a meal and drinks. Photovoice participants will receive CAD $20 (US $14.54) in cash at each meeting, along with snacks and drinks.

## Results

### Phase 1

Two preliminary meetings were held with a dozen collaborators. Representatives from 3 different community organizations working with people experiencing OUD, homelessness, or other marginalized conditions attended, along with 5 health care professionals who offer OAT services. The committee of peer researchers was formed in winter 2024, and the 10 meetings were held until June 2025.

### Phase 2

As of August 2025, 4 sessions had been completed with 12 individuals who are socially excluded and whose profiles correspond to those of the outreach clinic’s users. Preliminary results were presented at the 2023 North American Primary Care Research Group conference as part of a doctoral student project. In addition, a presentation on the initial impressions of using photovoice as an innovative research method to engage individuals who are socially excluded and use opioids was delivered at the 2024 International Society for the Study of Drug Policy conference. Analysis was completed in the spring of 2025.

### Phase 3

As of August 2025, 14 health care professionals and community workers had participated in the semistructured interviews. In addition, members of the peer researcher committee conducted 2 observation sessions at the outreach clinic in Longueuil. Preliminary results from the interviews were presented at the 2024 North American Primary Care Research Group conference as part of a medical resident project. Publication of the results of this phase, along with final and integrated results of phases 1 and 2, is expected in winter 2026.

Table 1 outlines the progression of the study.

**Table 1:** Study timeline.

Study phase	Time frame
Preparatory phase (preparatory meetings and interview guide)	Fall 2022
Phase 1: peer researcher committee	Fall 2023 through winter 2025
Phase 2: photovoice	Fall 2022 and fall 2023; analysis: winter 2025
Phase 3: interviews with professionals	Fall 2023 through winter 2025
Knowledge transfer phase	Spring 2024 through winter 2026

## Discussion

### A Service Adapted to User Needs

This project aims to evaluate the participatory implementation of an outreach clinic for OUD, with services adapted to the needs of its users. This innovative service offering will be coconstructed by involving people who are socially excluded and living with OUD (experiencing homelessness, with severe mental health disorders or in other vulnerable circumstances causing them difficulty in accessing basic addiction-related services), together with primary care or addiction care providers. The implementation of the primary care clinic with integrated OUD treatments that address overall health needs (eg, access to mental and physical health services, housing search assistance, and adapted employment integration) will be analyzed, and the service offering will be adapted to the needs of people living with OUD in the most marginalized conditions. This project will lay the foundation for a rigorous participatory evaluation process of this innovative intervention, following the example of another clinic in Montreal [52].

### Strategies to Ensure Rigorous Qualitative Research

The participatory approach presents specific challenges. Unequal power between the people involved in the study can lead to power and influence dynamics, often unconsciously [42], which can contribute to tensions and conflicts and undermine the participatory and collaborative nature of the approach [42,43,45]. Our research team will set up a committee made up exclusively of peers with current or past lived experience of OUD and will provide ongoing support to encourage their active participation in the project and the exploration of power-related topics [39,40,43]. Most of the partners in this project already have an established relationship of trust with the research team. We will also take the time to clarify the role and responsibility of each partner involved from the beginning of the research process [42] and then continually revalidate them with feedback and readjust them as necessary [42,45]. A logbook maintained by the research team will enable this process to be monitored.

The proposed research meets the rigorous criteria of qualitative research in a number of ways. First, with regard to the credibility and accuracy of the results, 3 coresearchers are involved in the clinic’s addiction treatment program, which enables the results to be enriched with nuances and extensive observations. After each interview, a debriefing between the research assistant performing the transcription and one of the coresearchers is scheduled to improve the process and enable verification of the interview report. In addition, after the initial analysis, a meeting is scheduled with the interview participants to verify the results of each interview and ensure the credibility of the interpretations. Second, to ensure the transferability of the results, the study team includes several coresearchers with clinical backgrounds. Their in-depth knowledge of the setting will enable a rich and detailed description of the research site. Although theoretical saturation may not be fully achieved, data redundancy is expected with the planned sample. Finally, the reliability of the results will be ensured by the careful application of procedures at each stage: during the interviews, using an interview guide and interview report; during transcription, performed by an experienced research assistant; and during analysis, with coding conducted by multiple research team members to enhance the neutrality of the interpretations.

### Conclusions

The results will provide decision-makers with coherent, evidence-based data for users and the community regarding the quality of care and services as well as their relevance and efficiency (improving value for money) and their ability to improve the health of the local population living with OUD and in precarious situations (improving population health). Thus, the process leading to the development of an outreach clinic for OUD outside major Canadian urban centers, as well as its subsequent evaluation and adaptation, will help improve health equity for all. The advantages of this project are that the intervention is coconstructed and monitored and that the evaluation process is initiated simultaneously. This project will provide useful data on the implementation of an addiction treatment program for people who are socially excluded in a semiurban context. Such data are essential for decision-makers because this population is the most difficult to reach in addiction treatment programs and has the least access to regular services due to multiple barriers [25,26]. This project could therefore help demonstrate to decision-makers the importance of developing such services in an appropriate way, particularly in the context of the dual crises of the COVID-19 pandemic and opioid overdoses.

In this project, the knowledge transfer strategy is based on the application of integrated knowledge. This approach is inherent to participatory research and enhances the potential for direct and immediate benefits for the organizations and partners involved. The community organizations, clinicians, and health care administrators involved will be able to apply the results in their respective environments. All team members will participate in disseminating the results. Peer researchers and people from community organizations will be called upon to present the results in dissemination activities.

## Appendix

Multimedia Appendix 1 Schedule of topics for peer-researcher committee.

Multimedia Appendix 2 Interview guide for professionals.

Multimedia Appendix 3 Peer review reports from the Catalyst Grant: Quadruple Aim and Equity Committee, Canadian Institutes of Health Research (CIHR).

## Abbreviations

OAT: opioid agonist therapy
OUD: opioid use disorder
