# Supplementary material for: Evaluation of the Implementation of an Outreach Clinic for Opioid Use Disorder: Protocol for a Participatory Cocreation and Implementation Study
Source: JMIR Res Protoc. 2025 Sep 18;14:e72457. doi: 10.2196/72457 (PMC12491895; doi:10.2196/72457)
Supplement: Multimedia Appendix 1 [file resprot_v14i1e72457_app1.docx]

**Appendix 1 : Schedule of topics for peer-researcher committee**

| Meeting | Topic / Objective |
| --- | --- |
| 1 | Presentation of the project and the committee members |
| 2 | Member checking meeting for the photovoice |
| 3 | Preparatory meeting for observation |
| 4 | Observations |
| 5 | Post-observation meeting |
| 6 | Working meeting on accessibility (availability, schedule, transportation, RAMQ, etc.) |
| 7 | Working meeting on safety (overdoses, safe supply, supervised consumption services, etc.) |
| 8 | Working meeting on clinic services (offered care, professionals, coordination with the healthcare network, coordination with community organizations, etc.) |
| 9 | Member checking meeting for the results of the 3 working meetings |
| 10 | Final meeting: messages, recommendations, future projects |
