# Supplementary material for: Evaluation of the Implementation of an Outreach Clinic for Opioid Use Disorder: Protocol for a Participatory Cocreation and Implementation Study
Source: JMIR Res Protoc. 2025 Sep 18;14:e72457. doi: 10.2196/72457 (PMC12491895; doi:10.2196/72457)
Supplement: Multimedia Appendix 2 [file resprot_v14i1e72457_app2.docx]

**Appendix 2 : Interview guide for professionals**

**Objectives**

1. **Evaluate the impacts of implementing a low-barrier, community-based opioid agonist treatment (OAT) clinic (STEP clinic) on professionals working in the addiction field**
   a. On patient care and follow-up
   b. On patients’ quality of life
2. **Assess potential challenges in referring patients to the STEP clinic**
   a. Awareness of the clinic
   b. Referral process
   c. Accessibility for the patient
3. **Evaluate the needs met or unmet by this type of clinic for healthcare professionals**
   a. In patient management
   b. For the patients themselves

**Questions**

- Content for a ~60-minute interview
- Include a brief explanation of the STEP clinic before questions 3–4–5 for professionals who are unfamiliar with it (see “STEP Clinic Explanation” document)

**SECTION 1: Candidate Profile**

1. Tell me a bit about yourself:
   - What is your current job?
   - What is your educational background?
   - What type of practice do you have, or what kind of clients do you work with?
   - Depending on your profile:
     - What led you to work in the field of addiction / homelessness / with vulnerable populations?

**SECTION 2: Impacts of the Clinic**

1. In your own words, explain how the STEP clinic operates and what services it offers.
   - How did you first hear about this clinic?
   - Do you have an idea of the type of patient profile the clinic is targeting?
2. Have you ever mentioned the STEP clinic to any of your patients who could benefit from care for opioid dependence?
   - What did you tell them? How did you introduce this option?
   - How did they respond? Were they interested? Any concerns?
   - How many patients have you referred to the STEP clinic?
   - In your opinion, how many of your patients could benefit from the STEP clinic’s services but don’t yet have access to them?
3. How has the implementation of the STEP clinic changed your daily practice?
   - Have you noticed any difference in your patients’ well-being?
   - If you were not previously aware of the STEP clinic: In what ways do you think it could change your practice?
   - What services could the STEP clinic offer to better support your work?

**SECTION 3: Evaluation of Challenges and Needs**

1. What obstacles have you encountered or do you anticipate when referring a patient to the STEP clinic?
   - How would you make a referral to the clinic?
   - What do you think would be the best method to facilitate referrals?
2. Do you have any suggestions to improve accessibility to the STEP clinic?
   - What do you think of the clinic’s location (between 3 CISSS, on Joliette Street in Longueuil, located in Virage’s facilities)?
   - What do you think of the clinic’s visibility (media, advertising)?
3. Do you have any positive or negative examples related to follow-up at the STEP clinic for your patients?
   - If not, do you have any positive or negative clinical situations involving OAT patients? In what ways could the STEP clinic have met those unmet needs?

**ADDITIONAL QUESTIONS:**

- In your opinion, what are patients looking for when they come to the STEP clinic?
  - Do you think the STEP clinic meets their needs?
  - Do you think patients clearly understand the different organizations and how they differ from the STEP clinic?
- What are your thoughts on the “low-barrier” approach of the clinic?
  - Are there any positive aspects for patient care?
  - Are there any barriers or drawbacks?
  - What differences do you see in follow-up care compared to clinics that are *not* low-barrier?
