# Supplementary material for: Evaluation of the Implementation of an Outreach Clinic for Opioid Use Disorder: Protocol for a Participatory Cocreation and Implementation Study
Source: JMIR Res Protoc. 2025 Sep 18;14:e72457. doi: 10.2196/72457 (PMC12491895; doi:10.2196/72457)
Supplement: Multimedia Appendix 3 [file resprot_v14i1e72457_app3.pdf]

Review Type/Type d'évaluation: Committee Member 1/Membre de comité 1
Name of Applicant/Nom du chercheur: Loignon, Christine
Application No./Numéro de demande: 475329
Agency/Agence: CIHR/IRSC
Competition/Concours: 2021-10-26 Catalyst Grant: Quadruple Aim and Equity/Subvention Catalyseur : Quatre objectifs et équité
Committee/Comité: Catalyst Grant : Quadruple Aim and Equity/Subvention catalyseur : Quatre objectifs et équité
Title/Titre: Co-construire une évaluation d'implantation d'une clinique de traitement des dépendances aux opioïdes à bas seuil d'accès, par et pour les pairs marginalisés utilisateurs d'opioïdes à Longueuil en Montérégie.

Assessment/Évaluation:

This project emphasizes the exacerbating effect of COVID-19 on the opioid crisis in Canada, as well as the lack of appropriate services to address it among marginalized people who use opioids. This is a patient-oriented project that is centred on empowerment and participatory management, where people who use opioids will be key team members. The project will bring together patient partners to co-construct a logical model describing a low barrier opioid addiction community clinic that they plan to opened in 2022 in Longueuil, Quebec. A committee of peers with opioid use disorders will be established, focus groups will be conducted with four groups: representatives of community organizations, stakeholders at municipal groups like police and the city, healthcare professionals, and peers with opioid use disorders. The focus groups will be co-facilitated by a researcher, a patient partner, and someone from a community organization. From this, a logic model for a multi-facetted service offer will be developed, taking into account the range of physical and mental health and social service needs of this clientele. As a second stage, a committee will conduct interviews with key stakeholders and review documents to study the feasibility of conducting a participatory action project to evaluate the implementation. They describe iterative qualitative data analysis methods for the various stages of the project, with triangulation among components.

Concept. The application is well written and clear. It presents an important idea and most of the outputs and outcomes are clearly defined.

Alignment. This project responds to a clear need in terms of developing patient-centred services for vulnerable people. By developing services outside of major urban centres, it addresses equity for underserved populations. The plan to work directly with people who use opioids to do this work would be expected to improve the patient experience and engagement in services, which, it could be hypothesized, would lead to better health outcomes and services that contain the components most important to patients, potentially supporting the cost/benefit ratio. The inclusion of providers in this work will support provider satisfaction in the services.

Equity, diversity and inclusion are not directly discussed in detail. While the team is targeting a vulnerable population, it would be helpful to hear how they will explicitly consider the impact of intersectional diversity on the services and on both the service users’ and service providers’ service preferences and experiences.

**Review Type/Type d'évaluation:**	Committee Member 1/Membre de comité 1
**Name of Applicant/Nom du chercheur:**	Loignon, Christine
**Application No./Numéro de demande:**	475329
**Agency/Agence:**	CIHR/IRSC
**Competition/Concours:**	2021-10-26 Catalyst Grant: Quadruple Aim and Equity/Subvention Catalyseur : Quatre objectifs et équité
**Committee/Comité:**	Catalyst Grant : Quadruple Aim and Equity/Subvention catalyseur : Quatre objectifs et équité
**Title/Titre:**	Co-construire une évaluation d'implantation d'une clinique de traitement des dépendances aux opioïdes à bas seuil d'accès, par et pour les pairs marginalisés utilisateurs d'opioïdes à Longueuil en Montérégie.

Assessment/Évaluation:

Feasibility. The work appears to be feasible within the timeframe of the grant. However, almost no attention is given to the implementation of the model, which they say they will do within the grant term. Implementation takes a lot of work and resources. The team might be underestimating the complexity of implementation of a newly generated model of service delivery. I think the proposed activities are strong and fit within the scope of this call, even if the implementation does not occur on schedule.

The leads are experienced in participatory action research and opioid addiction. The lead has a strong funding and publication track record in relevant subject areas and methods. Co-applicants bring complementary expertise and experience. There is some multidisciplinarity in the team. The team has a number of partnerships that may support feasibility and future funding. The principal knowledge user appears to have the ability to inform provincial decisions in the area of substance use, although they do not appear to be directly a decision maker themselves.

The team acknowledges the challenges that come with a participatory action approach, including the power dynamic, role confusion, and potential disagreements between stakeholders. The propose strong engagement practices as a mitigating strategy. COVID mitigating strategies are also proposed.

The description of the knowledge mobilization strategy is limited. The team does talk briefly about integrated knowledge translation and presentations to key stakeholders.

The budget consists of funding for a part-time research staff and two students, as well as interview transcription, some software, and compensation for participants, patient partners and community organization representatives. The budget is reasonable.

Potential impact. Co-construction of the model by people with lived experience will ensure that the constructed services are relevant to their experiences. The inclusion of service providers and other service stakeholders in the process will ensure that it also aligns with their needs within a realistic scope of

Review Type/Type d'évaluation: Committee Member 1/Membre de comité 1
Name of Applicant/Nom du chercheur: Loignon, Christine
Application No./Numéro de demande: 475329
Agency/Agence: CIHR/IRSC
Competition/Concours: 2021-10-26 Catalyst Grant: Quadruple Aim and Equity/Subvention Catalyseur : Quatre objectifs et équité
Committee/Comité: Catalyst Grant : Quadruple Aim and Equity/Subvention catalyseur : Quatre objectifs et équité
Title/Titre: Co-construire une évaluation d'implantation d'une clinique de traitement des dépendances aux opioïdes à bas seuil d'accès, par et pour les pairs marginalisés utilisateurs d'opioïdes à Longueuil en Montérégie.

Assessment/Évaluation:

programming in areas outside of major urban centres, where services are more scarce. They will have concrete service recommendations for knowledge users to guide service development. At the end of the project, they’ll also be ready to evaluate the resulting service, for ongoing improvement.The description of the knowledge mobilization strategy is limited. The team does talk briefly about integrated knowledge translation and presentations to key stakeholders, but it would be helpful to hear more about their plans. The principal knowledge user appears to have the ability to inform provincial decisions in this area, but is not a decision maker themselves.
